# Persistent unsealed internal limiting membrane after Nd:YAG laser treatment for valsalva retinopathy

**DOI:** 10.1186/1471-2415-13-15

**Published:** 2013-04-16

**Authors:** Ming Zou, Sheng Gao, Junjun Zhang, Meixia Zhang

**Affiliations:** 1 Department of Ophthalmology, West China Hospital of Sichuan University, No. 37 Guoxue Xiang, Wuhou District, Chengdu, Sichuan Province, 610041, China

**Keywords:** Internal limiting membrane, Nd:YAG laser membranotomy, Valsalva retinopathy

## Abstract

**Background:**

To report a long-term complication of unsealed and un-reattached internal limiting membrane in Valsalva retinopathy after neodymium-doped yttrium–aluminium–garnet (Nd:YAG) laser membranotomy.

**Case presentation:**

A 41-year-old man presenting with a massive premacular hemorrhage due to Valsalva retinopathy underwent Nd:YAG laser membranotomy. During follow-up, best-corrected visual acuity, retinal alteration and optical coherence tomography (OCT) outcomes were documented. One month after membranotomy, his visual acuity improved to 20/20 and the hemorrhage resolved completely. At an 8-month follow-up visit, the fundus showed progressive wrinkling of the internal limiting membrane with the laser perforation located in the center. OCT showed a persistent unsealed and un-reattached internal limiting membrane.

**Conclusions:**

Not all patients with Valslava retinopathy with premacular hemorrhage are appropriate candidates for laser membranotomy, especially patients with sub- internal limiting membrane hemorrhage. The key point for observation is the interface between internal limiting membrane and retinal surface. The long-term consequences of unsealed internal limiting membrane after Nd:YAG laser membranotomy require further understanding.

## Background

Valsalva retinopathy can present as a sudden, dramatic loss of central vision due to premacular hemorrhage [1]. While there is no widely accepted treatment modality other than observation, since the 1980s neodymium-doped yttrium–aluminium–garnet (Nd:YAG) laser membranotomy has been pushed to the forefront for the treatment of large macular hemorrhages in Valsalva retinopathy [2]. The membranotomy causes immediate drainage of the hemorrhage into the vitreous and away from the visual axis, prompting a rapid return of central visual acuity with few complications. The hemorrhage of Valsalva retinopathy may localize under the internal limiting membrane (ILM) and under the posterior hyaloid surface [3]. We report here a case of a patient with a persistent unsealed ILM after Nd:YAG laser treatment for Valsalva retinopathy with the hemorrhage under the ILM.

## Case presentation

**Figure 1:**
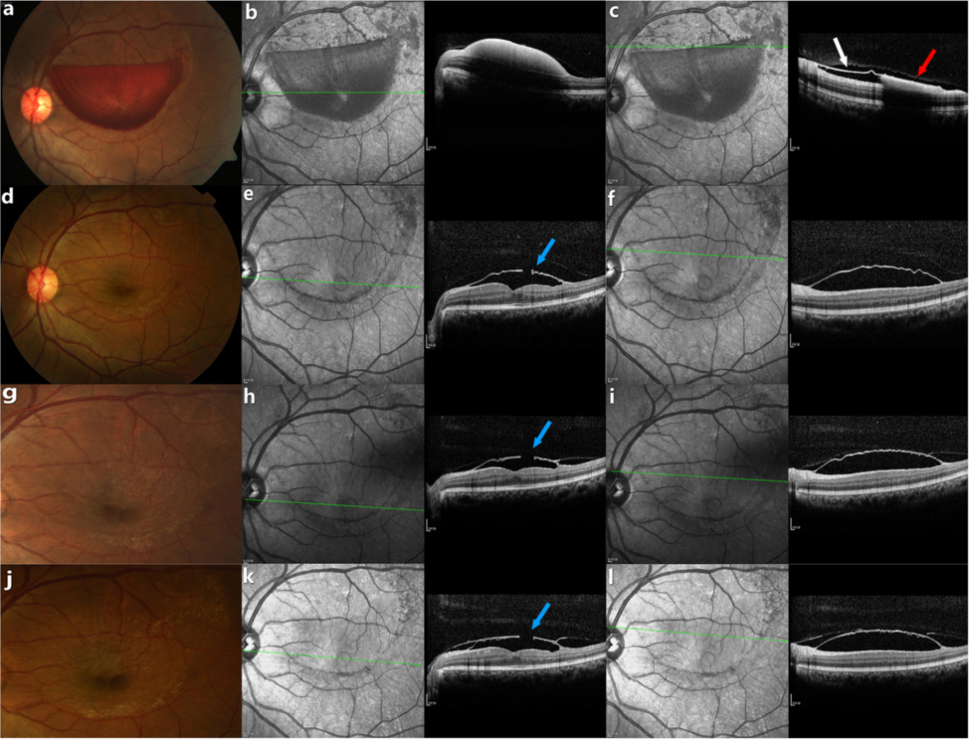
**Fundus photograph (a) of the left eye in a 41-year-old man with Valsalva retinopathy showing a massive premacular hemorrhage.** Corresponding SD-OCT reveals a dome-shaped hypo-reflective area consistent with blood (**b**). Above the level of settled blood there are two distinct membranes (**c**): the low reflective band of the posterior hyaloid surface (red arrow) and the hyper-reflective internal limiting membrane (ILM, white arrow). Fundus photography 1 month after laser (**d**) shows complete resolution of the hemorrhage. Corresponding SD-OCT shows the laser perforation in the ILM (**e**, blue arrow) and a sub-ILM hypo-reflective space (**f**). Fundus photography at 3 months (**g**) and 8 months (**j**) after the Nd:YAG laser membranotomy show progressive ILM wrinkling and striae radiating from the central perforation. Based on the OCT tracking system, the un-sealed ILM defect (blue arrow) is seen to persist at 3 months (**h**) and 8 months (**k**) after treatment; and the sub-ILM space also fails to show any substantial reduction in size at the 3-month (**i**) or 8-month (**l**) follow-up.

A 41-year-old man presented with sudden painless loss of vision in his left eye, occurring one morning after straining at stool because of constipation. Our patient had no history of vascular disease or blood dyscrasias. On ophthalmic examination, his left best-corrected visual acuity was hand motion at 5cm and his right visual acuity was 20/20. Retinal examination of the left eye showed a well-circumscribed premacular hemorrhage (Figure 1a). Spectral-domain optical coherence tomography (SD-OCT; Spectralis HRA+OCT, Heidelberg Engineering, Heidelberg, Germany) revealed a dome-shaped hypo-reflective area, consistent with blood beneath a hyper-reflective band at the macula (Figure 1b). Above the level of settled blood, two distinct membranes were shown: the patchy low reflectivity of the posterior hyaloid surface and the hyper-reflective internal limiting membrane (Figure 1c). We gave the patient three options to consider: observation alone, Nd:YAG laser membranotomy, or vitrectomy with release of the ILM. Considering the potential risks, benefits and costs of surgery and laser, at this stage the patient chose a conservative approach.

After 40 days of observation, his left visual acuity improved to counting fingers at 1 m. Fundus photograph revealed little resolution of the hemorrhage. A pulsed Nd:YAG laser membranotomy was performed (energy power 2.5 mJ; VISULAS YAG III, Carl Zeiss, Germany). The patient was followed-up monthly, and his visual acuity, retinal appearance and SD-OCT findings were documented.

After 1 month, visual acuity of the left eye had improved to 20/20 and the hemorrhage had been absorbed completely (Figure 1d). OCT revealed an unsealed gap and no reattachment of ILM (Figure 1e, 1f).

Three months after treatment, fundus photography demonstrated ILM striae, radiating from the central laser opening (Figure 1g). OCT showed that the ILM was still elevated with an un-sealed gap (Figure 1h, 1i).

At an 8-month follow-up visit, visual acuity was stable at 20/20 and the patient did not mention any symptoms of metamorphopsia. The fundus showed progressive wrinkling of the ILM (Figure 1j) in addition to a persistent unsealed and un-reattached ILM (Figure 1k, l). Over the course of subsequent follow-up visits, OCT tracking showed there to be no significant anatomic improvement of the laser perforation and no obvious resolution of the sub-ILM cavity.

## Conclusions

Nd:YAG lasers have been used for disruption of the posterior hyaloid or the ILM in the treatment of Valsalva retinopathy. The procedure proves to be effective in most patients within days and no laser-related complications are usually seen during the first 6 months [2-4].

The long-term complications of Nd:YAG laser membranotomy include macular hole [5], retinal detachment [5], epiretinal membrane formation [6] and a persistent premacular cavity [7]. The laser perforations and any elevations of the ILM usually seal and reattach within 2–6 months without any significant retinal changes [8]. However, in our case there was a persistent unsealed ILM after Nd:YAG membranotomy. As shown by OCT tracking: there was no significant reduction in the size of the ILM perforation or in the sub-ILM hyporeflective space.

Several causes may contribute to occur of the complication. First, a large perforating puncture of the ILM is unfavorable, although a larger opening may facilitate easier drainage of blood into the vitreous. Sealing of the perforation is by means of proliferating cells on the ILM and retinal surface [9]. For larger gap it may be harder to make contact between the layers, so making it difficult to seal by proliferative cells. Second, massive and long-term hemorrhages place the ILM under high tension, which can cause it to finally lose its elasticity. When tension persists for longer periods of time, the ILM may degenerate and cellular proliferation causes the formation of wrinkling. The resultant rigid tractional membrane is hard to reattach to the surface of retina. Finally, vitreous liquefaction induces the collection of fluid in the sub-ILM space, which also impedes apposition of the membrane and the closure of ILM defects.

In our case, the folds and striae of the ILM became increasingly worse over the course of the patients’ follow-up. The main cause of the patient’s poor prognosis was epiretinal membrane (ERM) formation. Kwok and colleagues (2003), reported a case with ERM secondary to Nd:YAG treatment, and there was a similar retinal outcome with our patient [6]. A persistent unsealed and un-reattached ILM membrane may drive ERM formation. To avoid this complication, the timing of the Nd:YAG laser procedure and the size of laser opening are important issues. Previous studies have shown that for patients with acute premacular hemorrhage, Nd:YAG membranotomy should be undertaken within the first 3 weeks, to avoid failure of drainage of blood into the vitreous. In our patient, it is possible that the presence of persistent hemorrhage beneath the ILM was the stimulus for ERM formation rather than the treatment itself.

In conclusion, puncturing the ILM by Nd:YAG laser is an effective procedure for Valsalva retinopathy where immediate clearance of hemorrhage is needed to restore vision. But not every patient is an appropriate candidate for laser, and the duration and amount of hemorrhage may be critical factors. The power setting required to make an appropriate puncture in the ILM is another important issue. During the follow-up after Nd:YAG laser membranotomy, assessing ILM alterations at the macular region is rather important and the long-term consequences of an unsealed ILM require further consideration.

### Consent

Written informed consent was obtained from the patient for publication of this case report and all accompanying images. A copy of the written consent is available for review by the Editor of this journal.

## Competing interests

The authors declare that they have no competing interests relevant to this article to disclose.

## Authors’ contribution

MXZ suggested this case report and participated in its development and coordination. MZ was the main physician responsible for the patient. MZ performed retinal examinations and OCT evaluation, and was involved in manuscript writing. SG performed the laser treatment and helped in reviewing literature sources for this manuscript. JJZ helped to draft the manuscript. All authors read and approved the final manuscript.

## Pre-publication history

The pre-publication history for this paper can be accessed here:

http://www.biomedcentral.com/1471-2415/13/15/prepub
